# Anesthetic Management of Total Aortic Arch Replacement in a Myasthenia Gravis Patient under Deep Hypothermic Circulatory Arrest

**DOI:** 10.1155/2019/3278147

**Published:** 2019-07-04

**Authors:** Mamiko Kondo, Yusuke Yoshikawa, Hirofumi Terada, Michiaki Yamakage

**Affiliations:** Department of Anesthesiology, Sapporo Medical University School of Medicine, South 1, West 16, Chuo-ku, Sapporo, Hokkaido 060-8543, Japan

## Abstract

The anesthetic management of myasthenia gravis patients undergoing cardiac or aortic surgery under cardiopulmonary bypass, especially with deep hypothermic circulatory arrest, is challenging. We describe a case of successful anesthetic management of a myasthenia gravis patient undergoing total arch replacement with deep hypothermic circulatory arrest under neuromuscular monitoring and complete reversal of the action of neuromuscular blocking drugs by sugammadex. The present case suggests that patients with well-controlled myasthenia gravis might be safely managed in cardiac or aortic surgery under cardiopulmonary bypass with deep hypothermic circulatory arrest.

## 1. Introduction

Myasthenia gravis (MG) is a rare autoimmune neuromuscular junction disorder mediated by antibodies against the acetylcholine receptor in the neuromuscular junction, and it can lead to a large variability in the sensitivity to neuromuscular blocking drugs [1, 2]. Careful use of neuromuscular blocking drugs is also required in cardiac and aortic surgery with cardiopulmonary bypass (CPB) since the pharmacokinetics and pharmacodynamics of neuromuscular blocking drugs can be altered during the hypothermic period of CPB and hypothermia itself decreases muscle strength [3, 4]. Moreover, deep hypothermic circulatory arrest (DHCA) substantially prolongs the effect of neuromuscular blocking drugs because of the extremely low body temperature. Hence, the anesthetic management of MG patients undergoing cardiac or aortic surgery under CPB, especially with DHCA, is challenging. Although some cases of successful anesthetic management for MG patients receiving cardiac surgery with CPB have been reported [5–8], there has been no report on anesthetic management of an MG patient who required CPB with DHCA. In this report, we describe a case of successful anesthetic management of an MG patient who received total arch replacement with DHCA. A written patient consent was obtained from the patient for publication purposes.

## 2. Case Report

A 71-year-old woman who weighed 59 kg was scheduled for total aortic arch replacement for treatment of a distal aortic arch aneurysm. The patient was primarily diagnosed with MG (Myasthenia Gravis Foundation of America Classification IIa) 3 years ago by a positive anti-acetylcholine receptor antibody and a positive edrophonium test. Since her MG was unresponsive to oral prednisolone and pyridostigmine and since thymoma was detected by computed tomography, thymectomy was performed a few months after diagnosis of MG. After thymectomy, her clinical symptoms significantly improved and oral prednisone was tapered to 6 mg daily. A physical examination conducted on admission to our hospital showed normal muscle strength.

On arrival in the operating room, an electrocardiogram, pulse oximeter, and noninvasive blood pressure monitoring were set up, and venous and radial artery catheters were inserted under local anesthesia. Rectal, bladder, and tympanic temperatures were monitored throughout surgery. Neuromuscular monitoring was recorded from the adductor pollicis muscle with train-of-four stimulation of the ulnar nerve using TOF-Watch (MSD, Japan). After preoxygenation with 100% oxygen, general anesthesia was induced with intravenous administration of 0.05 mg/kg midazolam and 2.5 *μ*g/kg fentanyl. TOF count of 0/4 was achieved by 0.5 mg/kg of rocuronium, followed by tracheal intubation without difficulty. After induction of general anesthesia, a transesophageal echocardiography probe and a central venous catheter were inserted. General anesthesia was maintained with inhalation of sevoflurane, continuous infusion of propofol, and intermittent administration of fentanyl and rocuronium. An additional 0.2 mg/kg of rocronium was administered after spontaneous neuromuscular recovery of T2 (second twitch of the train-of-four series). After median sternotomy, CPB was established with arterial perfusion via the ascending aorta and bicaval venous drainage. DHCA was achieved with selective antegrade cerebral perfusion at a tympanic temperature of 21°C. After the distal anastomosis was completed, DHCA was stopped and the systemic circulation was resumed through a side branch of the graft. Then, total aortic arch reconstruction was completed. The DHCA time and CPB time was 39 min and 146 min, respectively. Rocuronium was administered at total doses of 1 mg/kg, 0.4 mg/kg, and 0.2 mg/kg before, during, and after CPB, respectively. There was no patient movement throughout the procedure. Fentanyl was administered at a total dose of 20 *μ*g/kg during anesthesia. TOF count at the end of the surgery was 1/4 and the patient was transferred to the intensive care unit immediately after surgery. The patient gained consciousness and spontaneous ventilation was established shortly after administration of 200 mg of sugammadex. The trachea was extubated 5 hours after surgery. The patient's postoperative course was uneventful.

## 3. Discussion

There have been a few reports on perioperative anesthetic management of MG patients undergoing cardiac surgery with CPB [5–8]. Mild to moderate hypothermia was applied during CPB and nondepolarizing neuromuscular blocking drugs including atracurium, sisatracurium, and rocuronium were used in most of the cases. One patient was managed without any neuromuscular blocking drug during surgery [8]. The postoperative course was uneventful in all cases. However, this report is the first report on anesthetic management of an MG patient undergoing aortic surgery under CPB with DCHA. During DHCA, it is thought that the effects of neuromuscular blocking drugs will be substantially prolonged due to the decrease in metabolism caused by hypothermia. MG and deep hypothermia are expected to synergistically prolong the effects of neuromuscular blocking agents; however, our report indicates that MG patients can be safely managed under appropriate neuromuscular monitoring and the use of rocuronium and sugammadex even when they receive CPB with DHCA. Some previous reports showed that sugammadex can safely reverse rocuronium-induced neuromuscular blockade even in MG patients [9, 10]. However, consideration should be given to the fact that the MG in our patient was controlled well with only 6 mg/day of oral prednisolone after thymectomy. The clinical course might be different in patients with more severe MG.

The present case suggests that patients with well-controlled MG might be safely managed in cardiac or aortic surgery under CPB with DHCA. Appropriate neuromuscular monitoring and sugammadex for complete reversal of the action of neuromuscular blocking drugs could play crucial roles in the anesthetic management of such patients.
